# Association Between Lipoprotein (A) and Diabetic Nephropathy in Patients With Type 2 Diabetes Mellitus: A Meta-Analysis

**DOI:** 10.3389/fendo.2021.633529

**Published:** 2021-03-24

**Authors:** Xiaoyan Ren, Zhihui Zhang, Zhaoli Yan

**Affiliations:** Department of Endocrinology, Affiliated Hospital of Inner Mongolia Medical University, Hohhot, China

**Keywords:** lipoprotein (a), type 2 diabetes mellitus, diabetic nephropathy, observational studies, meta-analysis

## Abstract

**Background:**

Lipoprotein (a) [Lp (a)] has been well recognized as a risk factor of cardiovascular disease. However, the association between serum Lp (a) and diabetic nephropathy in patients with type 2 diabetes mellitus (T2DM) remains unknown. We performed a meta-analysis to comprehensively evaluate the above association.

**Methods:**

Observational studies aiming to evaluate the independent association between serum Lp (a) and diabetic nephropathy in T2DM patients were identified by systematic search of PubMed and Embase databases. A random-effect model which incorporated the potential intra-study heterogeneity was used for the meta-analysis.

**Results:**

Eleven observational studies with 9304 T2DM patients were included. Results showed that compared to those with the lowest Lp (a), patients with the highest Lp (a) level had higher odds of diabetic nephropathy (adjusted odds ratio [OR]: 1.63, 95% confidence interval [CI]: 1.25–2.14, I^2^ = 54%, P < 0.001). Meta-analysis of studies in which Lp (a) was presented as continuous variables showed consistent result (adjusted OR: 1.13 for 1 mg/dl increment of Lp (a), 95% CI: 1.03–1.24, I2 = 36%, P = 0.008). Subgroup analyses showed that study characteristics such as definitions of diabetic nephropathy and study design did not significantly affect the association (P for subgroup difference all > 0.05).

**Conclusions:**

Higher serum Lp (a) in patients with T2DM is independently associated with higher odds of diabetic nephropathy. Large scale prospective cohort studies are needed to validate this finding. Moreover, the potential influence of Lp (a) lowering on renal function in T2DM patients may be further investigated.

## Introduction

Patients with type 2 diabetes mellitus (T2DM) are vulnerable to kidney dysfunction, namely diabetic nephropathy (1). With the increasing incidence of DM globally, substantial patients with T2DM are suffering from diabetic nephropathy (2). As a common complication of T2DM, diabetic nephropathy has become one of the leading causes of end-stage renal disease (ESRD) all over the world (3). It has been reported that currently, over 20% of patients with diabetes ultimately develop diabetic nephropathy, which has become a major cause of mortality in these patients (4). Although various risk factors for diabetic nephropathy have been proposed, such as age, race, duration of diabetes, hyperglycemia, dyslipidemia, and hypertension etc., further recognition of residual risk factors for diabetic nephropathy remain of clinical significance for the risk stratification and management of the disease (5, 6).

Lipoprotein (a) [Lp (a)] has been well recognized as a risk factor of cardiovascular disease due to its atherogenic effects (7, 8). Lp (a) is a low-density lipoprotein (LDL)-like particle consisting of an apolipoprotein B100 (Apo B) molecule linked to a very large glycoprotein known as apolipoprotein (a), or apo (a) (7). Accumulating evidence suggests that higher serum Lp (a) may be associated with impaired renal function in populations (9). Although early study has proposed that higher serum Lp (a) may also be associated with renal dysfunction in diabetic patients, subsequent studies evaluating the association between Lp (a) and diabetic nephropathy showed inconsistent results (10, 11). Some studies suggested that serum Lp (a) is related to higher odds of diabetic nephropathy in T2DM patients (12–17), while others did not (18–22). Therefore, relationship between Lp (a) level and diabetic nephropathy remains undetermined. Accordingly, in this study, we performed a meta-analysis of observational studies to comprehensively evaluate the association between Lp (a) and diabetic nephropathy in patients with T2DM.

## Methods

The meta-analysis was performed in accordance with the MOOSE (Meta-analysis of Observational Studies in Epidemiology) (23) and Cochrane’s Handbook (24) guidelines.

### Literature Search

Studies were identified *via* systematic search of electronic databases of PubMed and Embase *via* the following terms: (1) “Lp(a)” OR “Lp (a)” OR “lipoprotein(a)” OR “lipoprotein (a)”; (2) “diabetes” OR “diabetic”; and (3) “renal” OR “kidney” OR “nephropathy” OR “proteinuria” OR “albuminuria” OR “nephropathies”. The search was limited to human studies published in English or Chinese. The reference lists of related original and review articles were also analyzed using a manual approach. The final literature search was performed on September 12, 2020.

### Study Selection

The inclusion criteria for the studies were: (1) observational studies published as full-length articles; (2) included adult patients with T2DM; (3) evaluated the association between serum Lp (a) and diabetic nephropathy; and (4) reported the relative risk for this association after adjustment of potential confounding factors. Definitions of diabetic nephropathy were in accordance with the definitions applied among the included studies, which typically include the presence of microalbuminuria (urinary albumin-creatinine ratio [ACR]: 30–299 μg/mg) or macroalbuminuria (urinary ACR ≥ 300 μg/mg), and/or reduced renal function as presented by the reduced estimated glomerular infiltrating rate (eGFR) or elevated serum creatinine (SCr) (1). Reviews, editorials, preclinical studies, and studies irrelevant to the aim of current meta-analysis were excluded.

### Data Extracting and Quality Evaluation

Literature search, data extraction, and quality assessment of the included studies were performed by two independent authors (XR and ZZ) according to the predefined inclusion criteria. Discrepancies were resolved by consensus. The extracted data included: (1) name of first author, publication year, and country where the study was performed; (2) study design characteristics; (3) participant characteristics, including health status, sample size, and sex; (4) patterns for Lp (a) analysis and cutoff values; (5) follow-up durations for cohort studies; (6) definitions of diabetic nephropathy; and (6) confounding factors adjusted in the multivariate analyses. The quality of each study was evaluated using the Newcastle-Ottawa Scale (25) which ranges from 1 to 9 stars and judges each study regarding three aspects: selection of the study groups; the comparability of the groups; and the ascertainment of the outcome of interest.

### Statistical Analyses

We used odds ratios (ORs) and their corresponding 95% confidence intervals (CIs) as the general measure for the association between Lp (a) and diabetic nephropathy in T2DM patients. For studies with Lp (a) analyzed as categorized variables, ORs of diabetic nephropathy in patients with the highest Lp (a) level compared to those with the lowest Lp (a) level were extracted. For studies with Lp (a) analyzed as continuous variables, ORs of diabetic nephropathy for each increment of 1mg/dl Lp (a) were extracted. Data of ORs and their corresponding stand errors (SEs) were calculated from 95% CIs or P values, and were logarithmically transformed to stabilize variance and normalized the distribution (24). For studies providing ORs with different adjusted factors, the ones with the most adequately adjusted factors were used in the meta-analysis. The Cochrane’s Q test and estimation of I^2^ statistic were used to evaluate the heterogeneity among the include cohort studies (26). A significant heterogeneity was considered if I^2^ > 50%. We used a random-effect model to synthesize the OR data because this model is considered as a more generalized method which incorporates the potential heterogeneity among the included studies (24). Sensitivity analyses, by omitting one individual study at a time, were performed to test the robustness of the results (27). Predefined subgroup analyses were performed to evaluate the influences of study characteristics on the outcome, including the definition of diabetic nephropathy, study design, and country of the study. The potential publication bias was assessed by visual inspection of the symmetry of the funnel plots. If more than 10 studies were included for each outcome, the Egger’s regression asymmetry test was further performed for the evaluation of potential publication bias (28). We used the RevMan (Version 5.1; Cochrane Collaboration, Oxford, UK) and STATA software (Version.12.0; Stata Corporation) for the meta-analysis and statistics.

## Results

### Literature Search

**Figure 1:**
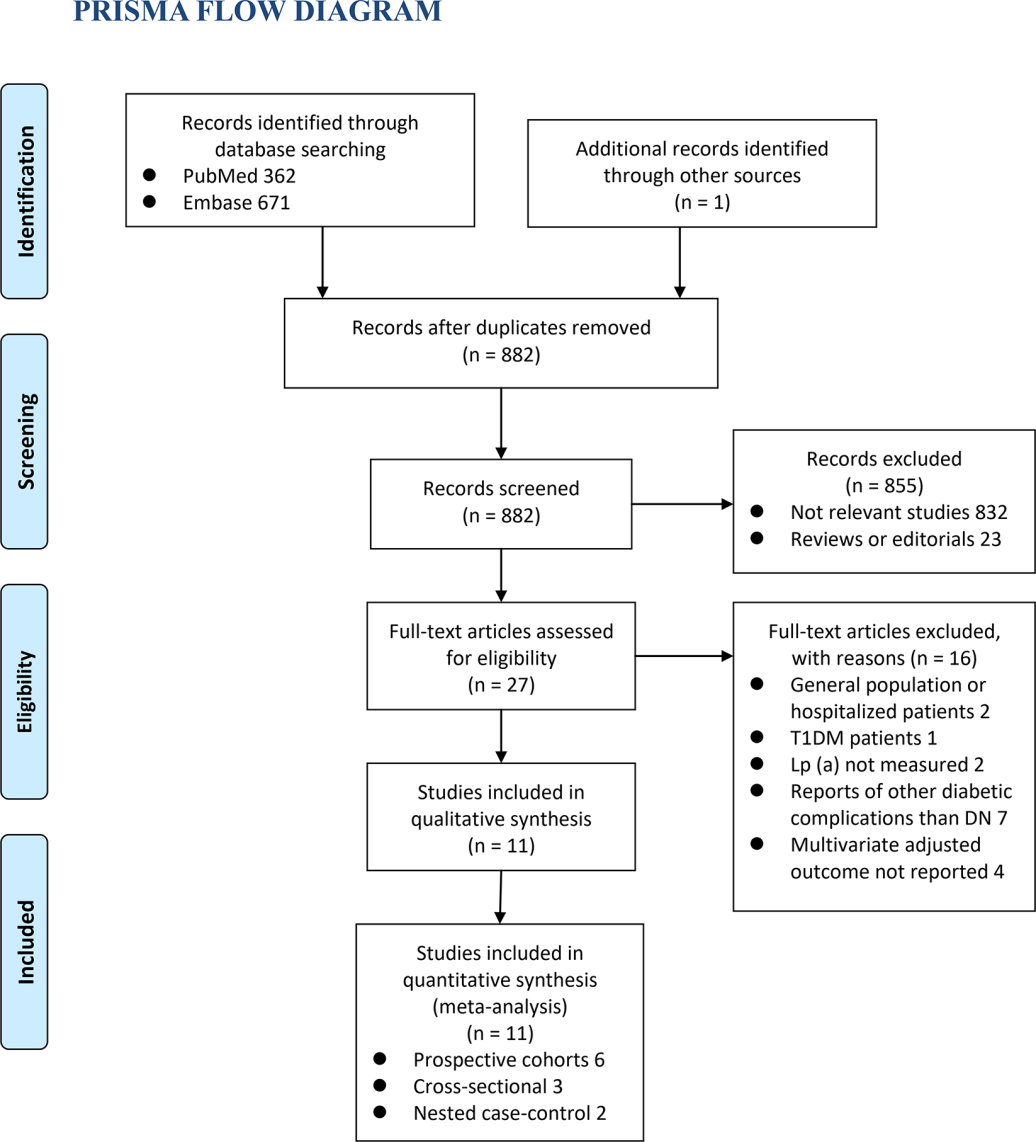
Flowchart of database search and study identification.

The process of database search was summarized in **Figure 1**. Briefly, 882 articles were found *via* initial literature search of the PubMed and Embase databases after excluding of the duplications. Among them, 855 were excluded through screening of the titles and abstracts mainly because they were not relevant to the purpose of the meta-analysis. Subsequently, 27 potential relevant records underwent full-text review. Of these, 16 were further excluded for the reasons listed in **Figure 1**. Finally, eleven observational studies, including six prospective cohort studies (12, 15, 17, 19, 21, 22), three cross-sectional studies (13, 14, 18), and two nested case-control studies (16, 20), were included into the meta-analysis.

### Study Characteristics and Quality Evaluation

The characteristics of the included studies were summarized in **Table 1**. Overall, eleven studies with 9304 T2DM patients were included. The studies were performed in Korea (12, 15), China (17, 18), Japan (14), Iran (16, 20), the United States (13, 19), Denmark (21), and the Netherlands (22). The mean ages of the patients varied from 53 to 69 years. The follow-up duration of the cohort studies varied from 2 to 11 years. In two studies, diabetic nephropathy was defined as the presence of microalbuminuria or macroalbuminuria (18, 22); in seven studies, diabetic nephropathy was defined as the decline of renal function as evidenced by reduced eGFR or elevated SCr (12, 13, 15–17, 19, 21); while in the other two studies, a combined outcome of albuminuria and/or decline of renal function was used (14, 20). Potential confounding factors, such as age, sex, smoking, body mass index, exercise, comorbidities, and use of antihypertensive medications, antidiabetic drugs, and lipid-lowering medications were adjusted to a varying degree. The NOS scores of the included studies ranged from seven to nine, indicating generally good study quality.

**Table 1:** Characteristics of the included observational studies.

Study	Country	Study design	Participants	Sample size	Mean age years	Male	Lp (a) presentation	Follow-up duration years	Definition of DN	Variables adjusted	Quality score
Song et al. (12)	Korea	PC	T2DM patients	81	59	44	Continuous	2	> 2-fold increase of follow-up SCr	Baseline SCr, SBP, and HbA1c	7
Tseng (18)	China	CS	T2DM patients	549	63	45	Continuous	NA	Microalbuminuria (ACR: 30–299 μg/mg) and macroalbuminuria (ACR ≥300 μg/mg)	Age, sex, BMI, diabetic duration, insulin use, SBP, use of statin/fibrate and use of ACEI/ARB	8
Lin et al. (19)	USA	PC	T2DM women	516	69	0	Q4 vs. Q1	11	eGFR decline of ≥ 25% during follow-up	Age, hypertension, BMI, ever smoked, physical activity, duration of T2DM, use of ACEI/ARB, baseline HbA1c and eGFR	9
Lin et al. (13)	USA	CS	T2DM patients without clinical CVD and with eGFR > 60 ml/min/1.73m^2^	1852	59	64	Continuous, and ≥ 30 mg/dl vs. < 30mg/dl	NA	eGFR: 60–90 ml/min/1.73m^2^	Age, sex, race, BMI, hypertension, lipid-lowering medications, HbA1c, HOMA-IR, duration on insulin, and urinary ACR	8
Yun et al. (15)	Korea	PC	T2DM patients with eGFR > 90 ml/min/1.73m^2^	560	53	40	T3 vs. T1	10	eGFR < 60 ml/min/1.73m^2^	Age, sex, diabetes duration, the presence of hypertension, CVD history, smoking, BMI, mean HbA1c, diabetic microvascular complication, FPG and Lp(a)-corrected LDL-C and medications like insulin, ACEI/ARB, statin, fenofibrate and acetylsalicylic acid	9
Senba et al. (14)	Japan	CS	T2DM patients	581	60	65	Above 90^th^ percentile vs. below 30^th^ percentile	NA	ACR ≥300 μg/mg and/or eGFR < 30 ml/min/1.73m^2^	Age, sex, BMI, HbA1c, duration of T2DM, current drinking, current smoking, hypertension, dyslipidemia, CAD, and stroke	8
Aryan et al. (20)	Iran	NCC	T2DM patients	939	58	48	Continuous, and Q4 vs. Q1	NA	Microalbuminuria (ACR: 30–299 μg/mg), macroalbuminuria (ACR ≥300 μg/mg), or eGFR < 60 ml/min/1.73m^2^	Age, sex, BMI, duration of diabetes, FPG, HbA1c, SBP, and the use of antihyperglycemic, antihypertensive and lipid-lowering medications	8
Singh et al. (22)	the Netherlands	PC	T2DM patients	1850	65	54	≥ 30 mg/dl vs. < 30mg/dl	7	Microalbuminuria (ACR: 30–299 μg/mg) and macroalbuminuria (ACR ≥300 μg/mg)	Age, sex, MAP, non-HDL-cholesterol, HDL-cholesterol, BMI, duration of type 2 diabetes, HbA1c and smoking	8
Heinrich et al. (21)	Denmark	PC	T2DM patients	198	59	74	Continuous	6	eGFR decline of ≥ 30% during follow-up	Age, sex, SBP, LDL-C, smoking, HbA1c, SCr and ACR	7
Xuan et al. (17)	China	PC	T2DM patients	1121	58	37	T3 vs. T1-2	4	eGFR < 60 ml/min/1.73m^2^	Age, sex, BMI, FPG, SBP, TG, HDL-C, LDL-C, eGFR, smoking and drinking status, and use of antihypertensive drugs and antidiabetic drugs	9
Moosaie et al. (16)	Iran	NCC	T2DM patients	1057	57	47	Continuous, and ≥ 34 mg/dl vs. < 34 mg/dl	NA	eGFR < 44 ml/min/1.73m^2^	Age, sex, SBP, HbA1c, BMI, use of anti-dyslipidemic drugs, eGFR, TG, LDL-C, HDL-C, non-HDL cholesterol, and waist/hip ratio	8

Lp (a), lipoprotein (a); DN, diabetic nephropathy; PC, prospective cohort; CS, cross-sectional; NCC, nested case-control; T2DM, type 2 diabetes mellitus; CVD, cardiovascular diseases; eGFR, estimated glomerular filtrating rate; Q, quartile; T, tertile; NA, not applicable; SCr, serum creatinine; ACR, albumin creatinine ratio; SBP, systolic blood pressure; HbA1c, glycated hemoglobulin; BMI, body mass index; ACEI, angiotensin converting enzyme inhibitor; ARB, angiotensin II receptor blocker; HOMA-IR, homeostasis model assessment of insulin resistance; FPG, fasting plasma glucose; LDL-C, low-density lipoprotein cholesterol; CAD, coronary artery disease; HDL-C, high-density lipoprotein cholesterol; TG, triglyceride; MAP, mean arterial pressure.

### Diabetic Nephropathy for Patients With Highest Versus Lowest Serum Lp (a) Levels

**Figure 2:**
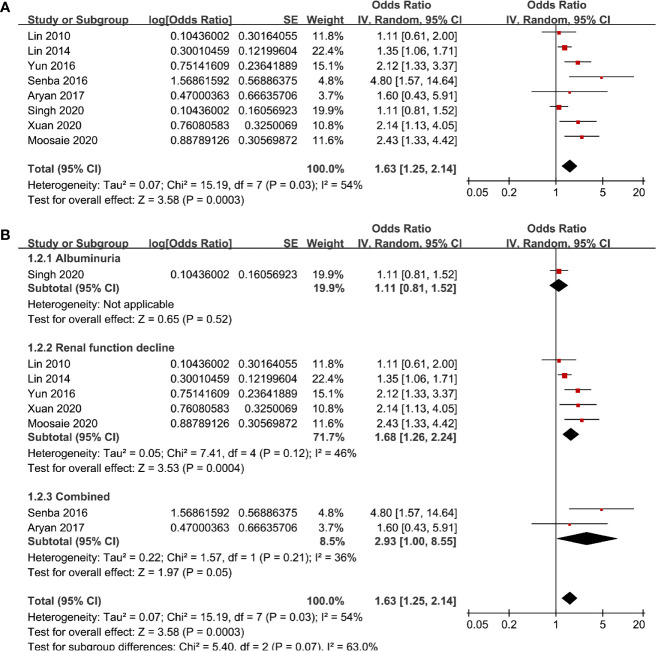
Forest plots for the meta-analysis of the association between Lp (a) analyzed as categorized variables and diabetic nephropathy in T2DM patients; **(A)** results of main meta-analysis; and **(B)** results of subgroup analyses according to definition of diabetic nephropathy.

**Figure 3:**
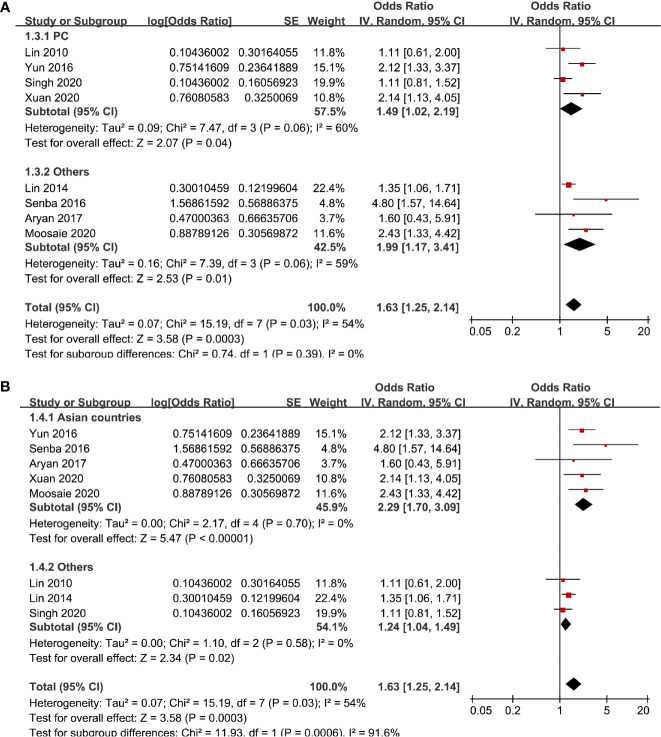
Subgroup analyses for the association between Lp (a) analyzed as categorized variables and diabetic nephropathy in T2DM patients. **(A)** Subgroup analyses according to the study design and **(B)** subgroup analyses according to the study country.

Eight studies (13–17, 19, 20, 22) evaluated the odds of diabetic nephropathy in T2DM patients with highest versus lowest serum Lp (a) levels. Pooled results with a random-effect model showed that patients with the highest Lp (a) level had higher odds of diabetic nephropathy (adjusted odds ratio [OR]: 1.63, 95% confidence interval [CI]: 1.25–2.14, I^2^ = 54%, P <0.001; **Figure 2A**). Sensitivity analysis by omitting one study at a time did not significantly change the results (OR: 1.54–1.80, P all < 0.05). Subgroup analysis showed that definition of diabetic nephropathy or study design did not significantly affect the association (both P for subgroup difference >0.05; **Figures 2B** and **3A**). However, the association between Lp (a) and higher odds of diabetic nephropathy were mainly driven by studies which defined diabetic nephropathy as decline of renal function (five studies, pooled OR: 1.68, 95% CI: 1.26–2.44, P <0.001). Moreover, the association between Lp (a) and diabetic nephropathy seemed to be stronger in studies from Asia (OR: 2.29, 95% CI: 1.70–3.09, P < 0.001) than that in studies from non-Asia (OR: 1.24, 95% CI: 1.04–1.49, P = 0.02; P for subgroup difference < 0.001; **Figure 3B**).

### Diabetic Nephropathy for the Increment of Serum Lp (a) of 1 mg/dl

**Figure 4:**
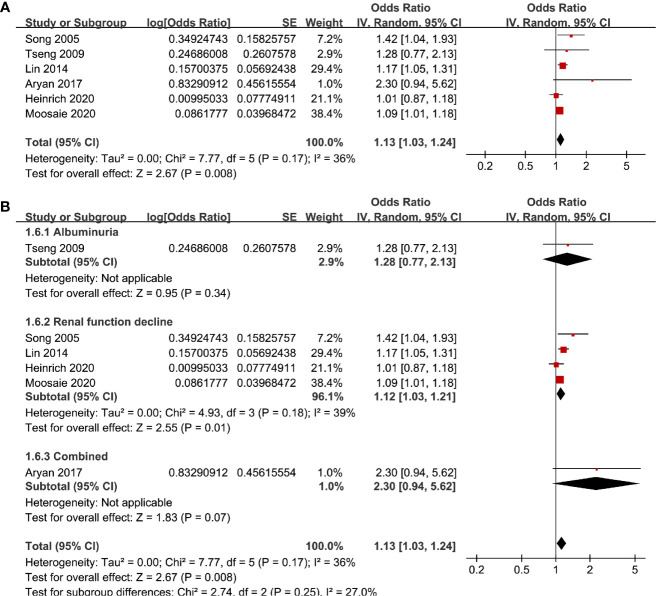
Forest plots for the meta-analysis of the association between Lp (a) analyzed as continuous variables and diabetic nephropathy in T2DM patients. **(A)** Results of main meta-analysis and **(B)** results of subgroup analyses according to definition of diabetic nephropathy.

**Figure 5:**
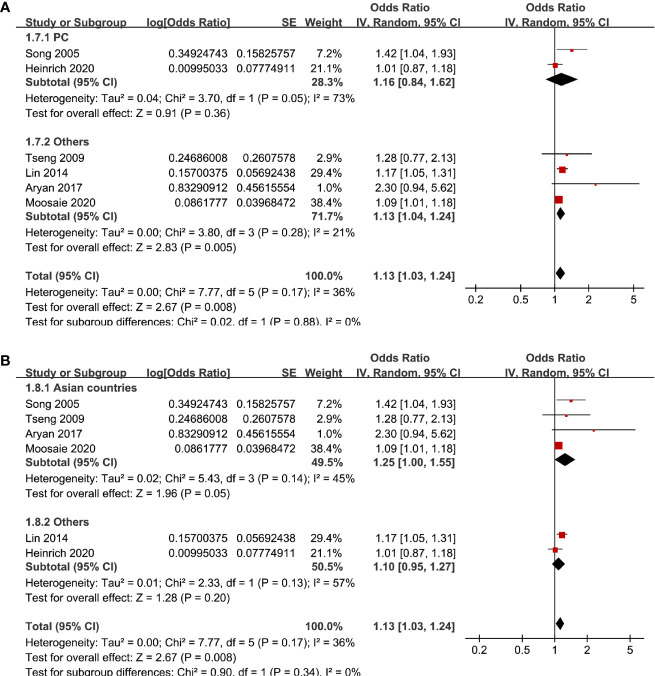
Subgroup analyses for the association between Lp (a) analyzed as continuous variables and diabetic nephropathy in T2DM patients. **(A)** Subgroup analyses according to the study design and **(B)** subgroup analyses according to the study country.

Six studies (12, 13, 16, 18, 20, 21) evaluated the odds of diabetic nephropathy in T2DM patients with serum Lp (a) as continuous variables. Pooled results with a random-effect model showed that higher serum Lp (a) was associated with higher odds of diabetic nephropathy (adjusted OR: 1.13 for 1 mg/dl increment of Lp (a), 95% CI: 1.03–1.24, I^2^ = 36%, P = 0.008; **Figure 4A**). Sensitivity analysis by omitting one study at a time did not significantly change the results (OR: 1.11–1.17, P all < 0.05). Subgroup analysis showed that definition of diabetic nephropathy, study design, or study country did not significantly affect the association (all P for subgroup difference > 0.05; **Figures 4B** and **5A, B**). Similarly, the association between Lp (a) and higher odds of diabetic nephropathy were mainly driven by studies which defined diabetic nephropathy as decline of renal function (four studies, pooled OR: 1.12, 95% CI: 1.03–1.21, P = 0.01).

### Publication Bias

**Figure 6:**
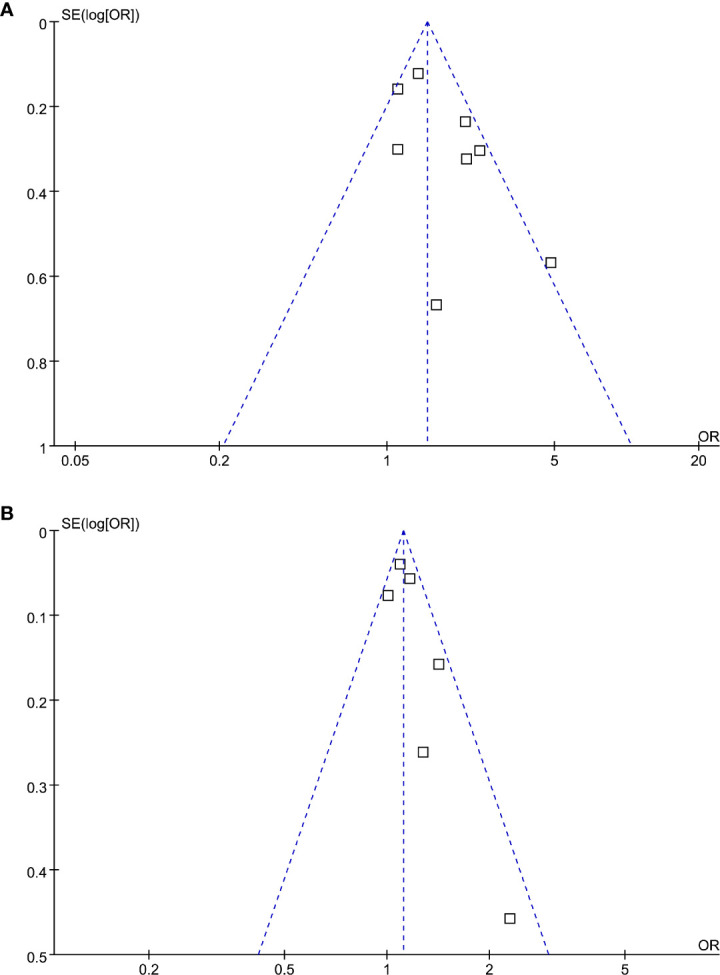
Funnel plots for the publication bias underlying the meta-analysis of the association between Lp (a) and diabetic nephropathy in T2DM patients. **(A)** Forest plots for the meta-analysis of studies with Lp (a) analyzed as categorized variables and **(B)** forest plots for the meta-analysis of studies with Lp (a) analyzed as continuous variables.

The funnel plots regarding the association between serum Lp (a) and diabetic nephropathy analyzed as categorized and continuous variables were shown in **Figures 6A, B**. The funnel plots were symmetry on visual inspection, suggesting low risk of publication bias. Egger’s regression tests were not performed since less than 10 datasets were available for each meta-analysis.

## Discussion

This meta-analysis of observational studies showed that higher serum Lp (a) was associated with increased odds of diabetic nephropathy in patients with T2DM. The association between Lp (a) and diabetic nephropathy were consistent in studies with Lp (a) analyzed as categorized or continuous variables. Results of subgroup analyses suggested that the association between Lp (a) and diabetic nephropathy may not be significantly affected by the differences of the definitions of diabetic nephropathy or study design. However, the association between Lp (a) and diabetic nephropathy seemed to be stronger in studies from Asia than that in studies from non-Asia. Taken together, these results demonstrated that higher serum Lp (a) may be independently associated with higher odds of diabetic nephropathy in patients with T2DM. Although further large-scale prospective cohort studies are needed to confirm these findings, the potential influence of Lp (a) lowering on renal function in T2DM patients may be investigated in future studies.

To the best of our knowledge, this study is the first meta-analysis to summarize the relationship between serum Lp (a) and diabetic nephropathy in T2DM patients. The strengths of the meta-analysis may include the following. Firstly, the finding that Lp (a) is associated with diabetic nephropathy was based on multivariable adjusted data, indicating that the above association may be independent of potential confounding factors, such as age, sex, smoking, obesity, comorbidities, and concurrent medications. These results may suggest an independent association between Lp (a) and diabetic nephropathy. Secondly, studies with Lp (a) analyzed as categorized and continuous data were summarized separately and derived consistent results, which further validated the robustness of the meta-analysis. Thirdly, sensitivity analyses by omitting one study at a time did not significantly affect the results, suggesting the stability of the finding. Fourthly, multiple subgroup analyses were performed to evaluate the potential study characteristics on the association between Lp (a) and diabetic nephropathy. Although limited datasets were included for some stratum and interpretation of subgroup results should be cautiously, findings of subgroup analyses may be clinically relevant. Subgroup analysis did not show that differences in the definitions of diabetic nephropathy may significantly affect the results. However, the significant association between Lp (a) and diabetic nephropathy were mainly driven by studies with diabetic nephropathy defined as renal function decline. These findings suggests that higher Lp (a) may more likely to be associated with late changes of diabetic nephropathy evidenced by increased SCr or reduced eGFR (9). An early study in patients with chronic kidney disease (CKD) showed a rapid decrease of Lp (a) levels after renal transplantation, but not after initiation of hemodialysis, which suggested that the increase in Lp (a) seen in CKD is due to loss of functioning renal tissue (29). Moreover, a previous cohort study in T2DM patients without CKD at baseline also showed that baseline Lp (a) levels >30 mg/dl were associated with a decline in eGFR by 2.75 mL/min/year compared to 1.01 mL/min/year in subjects with baseline Lp(a) less than 30 mg/dl, which is consistent with our findings (30). In addition, we found that the association between Lp (a) and diabetic nephropathy seemed to be stronger in studies from Asia than that in studies from non-Asia. Previous studies have suggested the potential ethnic differences in the optimal cut-off values of Lp (a) (31) and its association with CVD risks (32, 33). Interestingly, an early cross-sectional study in the US population also showed that a low eGFR is associated with moderately greater Lp (a) levels in a race-ethnicity different manner (34). Future studies are needed to confirm whether an ethnic difference exists regarding the association between serum Lp (a) and diabetic nephropathy in T2DM patients.

The mechanisms underlying the potential association between Lp (a) and diabetic nephropathy may be multifactorial. The most likely explanation is that serum Lp (a) levels reflect a balance of Lp (a) synthesis in the liver and catabolism possibly involving kidney (9, 35). As previously mentioned, in patients with ESRD, Lp (a) levels were significantly increased compared to healthy controls, which were rapidly decreased after kidney transplantation but not after the initiation of hemolysis (29). These findings may support a metabolic role of the kidney in Lp (a) catabolism and suggest that the increase in Lp (a) seen in CKD is probably due to loss of functioning renal tissue (29). Besides, it has also been suggested that the increase in Lp (a) associated with protein-losing related renal disease is likely to be a result of a general increase in protein synthesis by the liver due to high urinary protein loss rather than decreased catabolism (36). Currently, it remains unclear whether increased Lp (a) plays key roles in the pathogenesis of diabetic nephropathy or it is just a marker of impaired renal function. In view of the atherogenic role of Lp (a) and the importance of glomerular atherosclerosis in the pathogenesis of diabetic nephropathy (11), Lp (a) may be involved in the progression of diabetic nephropathy *via* its atherogenic effect. An early study *in vitro* study showed that low concentrations of Lp (a) stimulated growth of mesangial cells, whereas higher concentrations had antiproliferative or toxic effects, which may both have a negative impact on the course of renal disease (37). Another study showed that Lp (a) stimulated the growth of human mesangial cells and induced the activation of phospholipase C, which may therefore contribute to pathophysiology of renal disease (38). Moreover, oxidative stress has been confirmed to play a key role in the pathogenesis of diabetic renal complications (39, 40). Lp (a) was reported to induce the generation of oxygen-free radicals *in vitro*, which may partly contribute to kidney injury in diabetes (41). Besides, Lp(a) is susceptible to oxidative modification, leading to extensive formation of pro-inflammatory oxidized phospholipids, oxysterols, oxidized lipid-protein adducts in Lp(a) particles, which may perpetuate kidney injury (42). Future studies are needed to determine the possible pathophysiological mechanisms underlying the association between Lp (a) and diabetic nephropathy, and the potential influence of Lp (a) lowering on renal function in T2DM patients may also be investigated

Our study has limitations which should be considered when the results were interpreted. Firstly, considerable heterogeneity was detected among the included studies. Although we performed subgroup analysis to explore the potential influences of study characteristics such as definitions of diabetic nephropathy, study design, and study country, other factors may also contribute to the heterogeneity. Specifically, dietary natural products and medications that may affect serum Lp (a) are likely to modify the association between Lp (a) and diabetic nephropathy, such as phytosterol (43, 44), flaxseed (45), L-carnitine (46), and various lipid-lowering medications (47–49), which were rarely reported in the included studies. In addition, outcome of diabetic nephropathy should be optimally reported according to the stages of the disease (1). However, since various definitions of diabetic nephropathy was applied within the included studies, and none of them reported the outcome according to the stage of the disease, we were unable to determine the association between Lp(a) and diabetic nephropathy according to the disease stage. Besides, since the definitions of diabetic nephropathy within the included studies mainly focused on albuminuria and decreased eGFR, two key features of diabetic nephropathy (1), results of our study could reflection the association between Lp(a) and diabetic nephropathy. We have acknowledged this as a limitation of current meta-analysis, and future studies are needed to determine the association between Lp(a) and diabetic nephropathy of different disease stages. Moreover, since the individual patient data was not available, we could only perform subgroup analyses based on study-level data. In addition, in view of the limited datasets available for subgroup analyses, the results of subgroup analyses should be interpreted with caution. Furthermore, although we included studies with adjusted data, we could not exclude the existence of residual factors which may confound the association. Besides, it remains unknown whether the association is linear, or what the optimal cutoff value of Lp(a) is as for the prediction of diabetic nephropathy. Results of meta-analysis highlighted the importance of further studies (prospective cohort studies with large sample size) to investigate these issues. Finally, a causative association between higher serum Lp (a) and increased odds of diabetic nephropathy in T2DM patients should not be derived based on our finding since this study was a meta-analysis of observational studies.

In conclusion, higher serum Lp (a) in patients with T2DM is independently associated with higher odds of diabetic nephropathy. Large scale prospective cohort studies are needed to validate this finding, and the potential influence of Lp (a) lowering on renal function in T2DM patients may be further investigated.

## Data Availability Statement

The original contributions presented in the study are included in the article/supplementary material. Further inquiries can be directed to the corresponding author.

## Author Contributions

XR and ZY designed the study. XR and ZZ performed literature search, data extraction, and quality evaluation. XR and ZY performed statistical analyses. XR wrote the manuscript. All authors reviewed and revised the manuscript, and approved the manuscript for submission. All authors contributed to the article and approved the submitted version.

## Funding

This study was supported by the Major Scientific Research Program of the Affiliated Hospital of Inner Mongolia Medical University (NYFY ZD 001).

## Conflict of Interest

The authors declare that the research was conducted in the absence of any commercial or financial relationships that could be construed as a potential conflict of interest.
